# Supplementary material for: New Drinking Water Genome Catalog Identifies a Globally Distributed Bacterial Genus Adapted to Disinfected Drinking Water Systems
Source: Environ Sci Technol. 2024 Sep 5;58(37):16475–87. doi: 10.1021/acs.est.4c05086 (PMC11411728; doi:10.1021/acs.est.4c05086)
Supplement: Supplementary file 1 — es4c05086_si_001.pdf [file es4c05086_si_001.pdf]

SUPPORTING INFORMATION

New drinking water genome catalog identifies a globally distributed bacterial genus adapted to disinfected drinking water systems

Ashwin S Sudarshan[1], Zihan Dai[1], Marco Gabrielli[2], Solize Oosthuizen-Vosloo[3], Konstantinos T. Konstantinidis[1], Ameet J Pinto[1,4*]

[1] School of Civil and Environmental Engineering, Georgia Institute of Technology, Atlanta, USA

[2] Department of Environmental Microbiology, Eawag, Swiss Federal Institute of Aquatic Science and Technology, Dubendorf, Switzerland,

[3] Institute for Cellular and Molecular Medicine, Department of Immunology, Faculty of Health Sciences, University of Pretoria, Pretoria, South Africa

[4] School of Earth and Atmospheric Sciences, Georgia Institute of Technology, Atlanta, USA

*Corresponding authors: ameet.pinto@ce.gatech.edu

Supporting information: Four pages, eight tables, and two figures.

Supplementary Table S1: (A) Details of metagenomes used to construct the DWGC. (B) Accession numbers for isolate genomes obtained from NCBI and their isolation sources (see Excel spreadsheet).

Supplementary Table S2: SILVA v138.1 Phreatobacter sequences used for comparative analysis (see Excel spreadsheet).

Supplementary Table S3: Summary of metagenome assembly and bins across different distribution systems (see Excel spreadsheet).

Supplementary Table S4: Detailed taxonomy and genomic information for 1141 MAGs within the DWGC. Taxonomy was estimated using GTRDB (release 207_v2), completeness/redundancy were estimated using CheckM2, while presence/absence of tRNAs/rRNA genes was determined using Prokka v1.14.6 (see Excel spreadsheet).

Supplementary Table S5: Relative Abundance of Species across the 80 distribution systems used to determine the core microbiome (see Excel spreadsheet).

Supplementary Table S6: Detection frequency and average relative abundance of genera in the DWGC (see Excel spreadsheet).

Supplementary Table S7: Metabolism annotation results for Lineage 1 (L1) and Lineage 2 (L2) MAGs (see Excel spreadsheet).

Supplementary Table S8: Complete list of names proposed in the current register SeqCode list.

**Proposed Taxon**	**Etymology**	**Description**	**Parent Taxon**	**Type**	**Registry URL**
Genus Raskinella	[Ras.ki.ne'lla] **N.L. fem. n.** Raskin, Derived from Dr. Lutgarde Raskin; **N.L. fem. dim. n.** Raskinella, Named after Dr. Lutgarde Raskin	Raskinella is a novel genus under the family Phreatobacteraceae and order Rhizobiales. Organisms within this genus were identified in disinfected drinking water distribution systems.	*Phreatobacteraceae*	Raskinella chloraquaticus^{Ts}	seqco.de/i:46679
Species Raskinella chloraquaticus^{Ts}	[chlor.a.qua'ti.cus] **N.L. neut. n.** chlorum, chlorine; **L. masc. adj.** aquaticus, of or from water; **N.L. masc. n.** chloraquaticus, relating to chlorinated water	Raskinella Chlorumaquaticus is a globally prevalent and abundant species that has been identified within disinfected drinking water systems. It is part of a unclassified genus "Raskinella" under the family Phreatobacteraceae.	*Raskinella*	NCBI Assembly: GCA_002365175.1^{Ts}	seqco.de/i:46680

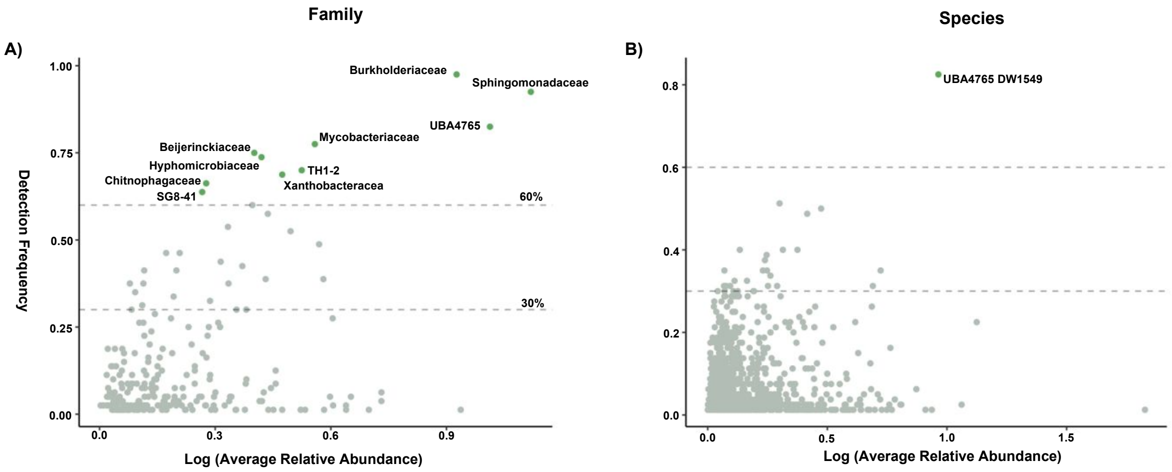

Supplementary Figure S1: A) Detection Frequency Vs Log of Average Relative abundance of the DWGC genomes at Family level. B) Detection Frequency Vs Log of Average Relative abundance of the DWGC genomes at Species level.

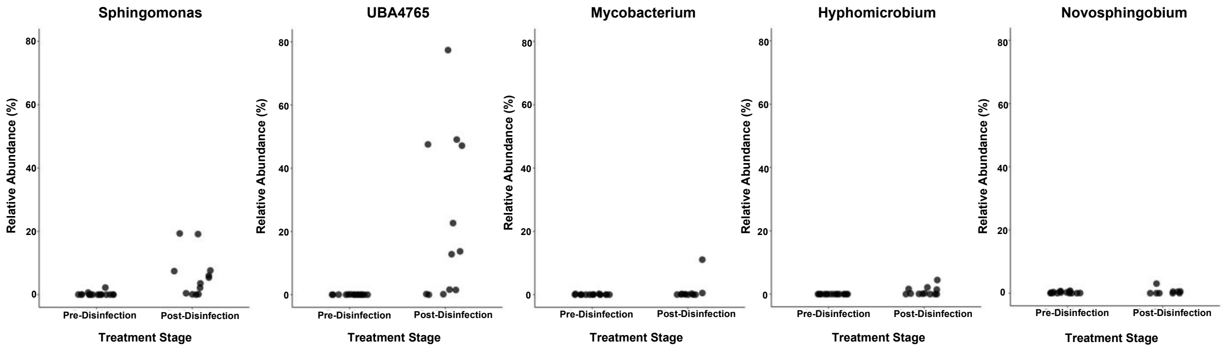

Supplementary Figure 2: Relative abundance of MAGs from five genera observed in more than 60% of the systems Pre- and Post-disinfection in studies where Pre-disinfection metagenomes were available.
